# Pharmacodynamics and cellular accumulation of amphotericin B and miltefosine in *Leishmania donovani*-infected primary macrophages

**DOI:** 10.1093/jac/dky014

**Published:** 2018-02-28

**Authors:** Andrew A Voak, Joseph F Standing, Nuno Sepúlveda, Andy Harris, Simon L Croft, Karin Seifert

**Affiliations:** 1 Faculty of Infectious and Tropical Diseases, Department of Immunology and Infection, London School of Hygiene & Tropical Medicine, London WC1E 7HT, UK; 2 Great Ormond Street Institute of Child Health, University College London, London WC1N 1EH, UK; 3 Centre for Statistics and Applications of University of Lisbon, Lisbon, Portugal; 4 Pharmidex, 3rd Floor, 14 Hanover Street, London W1S 1YH, UK

## Abstract

**Objectives:**

We examined the *in vitro* pharmacodynamics and cellular accumulation of the standard anti-leishmanial drugs amphotericin B and miltefosine in intracellular *Leishmania donovani* amastigote–macrophage drug assays.

**Methods:**

Primary mouse macrophages were infected with *L. donovani* amastigotes. In time–kill assays infected macrophages were exposed to at least six different concentrations of serially diluted drugs and the percentage of infected macrophages was determined after 6, 12, 24, 48, 72 and 120 h of exposure. Cellular drug accumulation was measured following exposure to highly effective drug concentrations for 1, 6, 24, 48 and 72 h. Data were analysed through a mathematical model, relating drug concentration to the percentage of infected cells over time. Host cell membrane damage was evaluated through measurement of lactate dehydrogenase release. The effect of varying the serum and albumin concentrations in medium on the cellular accumulation levels of miltefosine was measured.

**Results:**

Amphotericin B was more potent than miltefosine (EC_50_ values of 0.65 and 1.26 μM, respectively) and displayed a wider therapeutic window *in vitro*. The kinetics of the cellular accumulation of amphotericin B was concentration- and formulation-dependent. At an extracellular concentration of 10 μM miltefosine maximum cellular drug levels preceded maximum anti-leishmanial kill. Miltefosine induced membrane damage in a concentration-, time- and serum-dependent manner. Its cellular accumulation levels increased with decreasing amounts of protein in assay medium.

**Conclusions:**

We have developed a novel approach to investigate the cellular pharmacology of anti-leishmanial drugs that serves as a model for the characterization of new drug candidates.

## Introduction

The leishmaniases are neglected tropical diseases, caused by parasites of the genus *Leishmania*. In the human host, parasites survive and multiply as intracellular amastigotes in the parasitophorous vacuole of primarily tissue-resident macrophages.^1^ Disease manifestations include cutaneous leishmaniasis, mucocutaneous leishmaniasis and visceral leishmaniasis (VL).^2^^,^^3^ Based on recent estimates the leishmaniases are endemic in at least 98 countries and there are 0.7–1.2 million cutaneous leishmaniasis and 0.2–0.4 million VL cases each year. The number of deaths attributed to VL is estimated at 20 000–40 000 per year.^4^ There is currently no vaccine licensed for human use and available drug treatments have limitations.^5^^,^^6^

Pharmacokinetics/pharmacodynamics (PK/PD) strives to understand the relationship between drug concentrations and biological effects. Cellular PK is centred on the evaluation of penetration, distribution, degradation and efflux of drugs in individual cells and has been widely applied to research on antibiotics.^7^ In the case of intracellular infections, cellular PK is an important determinant of anti-infective drug action as it describes processes and exposure at the site of infection, previously limited for anti-leishmanial drugs.

Here we determined the *in vitro* PD and cellular accumulation of two VL drugs, miltefosine and amphotericin B, as both the deoxycholate salt (Fungizone^®^) and liposomal formulation (AmBisome^®^),^5^^,^^6^ in primary mouse macrophages infected with *Leishmania donovani*.

## Materials and methods

### Reagents and anti-leishmanial drug stocks

RPMI 1640 medium, DMEM, l-glutamine, acetonitrile, DMSO, tolbutamide, fatty acid-free BSA, Dulbecco’s PBS (DPBS), PBS and penicillin/streptomycin were purchased from Sigma (UK). Heat-inactivated FBS (hi-FBS), 0.1% (v/v) formic acid in water (LC-MS grade), methanol (HPLC grade), water (LC-MS grade), BSA fraction V and LIVE/DEAD^®^ stain were purchased from Fisher Scientific (UK). Flow Cytometry Staining Buffer was purchased from eBioscience (UK).

Amphotericin B deoxycholate (Fungizone^®^) was purchased from University College London Hospitals (UK). A 5.4 mM stock solution was prepared according to the manufacturer’s instructions. Liposomal amphotericin B (AmBisome^®^) was purchased from Gilead (UK) and the powder reconstituted following the manufacturer’s directions. Miltefosine was obtained from Paladin Labs Inc. (Montreal, Canada). A 20 mM stock solution was prepared as described previously.^8^

### Host cells and infection

Bone marrow-derived macrophages (BMDMs) were obtained from femurs of female BALB/c mice, aged 6–11 weeks, as described previously.^8^ Briefly, bone cavities were flushed with DMEM plus 10% hi-FBS, 100 U/mL penicillin and 100 mg/L streptomycin. Cells were pelleted by centrifugation (1400 rpm, 10 min, 4°C) and re-suspended in the above medium plus 20% L-929 fibroblast culture supernatant (source of macrophage colony-stimulating factor). The suspension was incubated in Petri dishes at 37°C/5% CO_2_ for 6 days with the addition of fresh medium after 3–4 days. Following replacement of medium with ice-cold PBS and incubation on ice, macrophages were gently dislodged with a rubber cell scraper and harvested by centrifugation at 1500 rpm for 10 min at 4°C. Macrophages were re-suspended in RPMI 1640 medium + 10% hi-FBS and plated in 16-well chamber slides (Fisher Scientific, UK) at a density of 4 × 10^4^ macrophages/well (for PD and cytotoxicity experiments) or in 4-well chamber slides (Fisher Scientific, UK) at a density of 2.5 × 10^5^ macrophages/well (for cellular accumulation experiments). After 8 h of incubation at 37°C/5% CO_2_*L. donovani* amastigotes (strain MHOM/ET/67/HU3 or strain MHOM/Sudan/09/SUKA001), freshly harvested from the spleen of a Rag-1-knockout (B6) mouse [London School of Hygiene and Tropical Medicine (LSHTM) breeding colony] and re-suspended in RPMI 1640 medium + 10% hi-FBS, were added at a ratio of 10 amastigotes/1 macrophage.

Mouse peritoneal exudate cells (PECs) were harvested from female BALB/c mice (LSHTM breeding colony) after intraperitoneal injection of 2% soluble starch as described previously^8^ and plated in RPMI 1640 medium + 10% hi-FBS in 16-well chamber slides at 4 × 10^4^ macrophages/well. Cultures were incubated overnight at 37°C/5% CO_2_ and infected the next day as described above.

Host cells were coincubated with amastigotes overnight at 37°C/5% CO_2_ and non-phagocytosed amastigotes washed off the next day, prior to processing cell cultures for further experiments as described below.

### Host cell surface marker analysis

BMDMs and PECs were suspended in a 5% (w/v) solution of BSA fraction V in Flow Cytometry Staining Buffer at 2 × 10^7^ cells/mL. The suspension was incubated on ice for 20 min and then mixed with an equal volume (50 μL) of antibodies, diluted in Flow Cytometry Staining Buffer. This suspension was incubated for 45 min on ice in the dark prior to addition of 2 mL of ice-cold PBS and centrifugation at 1000 rpm for 5 min at 4°C. The pellet was resuspended in 0.1% LIVE/DEAD^®^ stain in ice-cold PBS and incubated on ice for 30 min. Cells were washed three times in Flow Cytometry Staining Buffer and resuspended in 1 mL of Flow Cytometry Staining Buffer, followed by filtration through a 40 μm nylon mesh. Cell suspensions were collected in pre-cooled FACS tubes, protected from light. Cells were acquired on an LSR II flow cytometer (BD Biosciences, UK) and data analysis performed using FlowJo analytic software (Treestar, USA). Fluorescence Minus One and isotype controls were included. Antibodies and final dilutions used for surface staining were CD11b-FITC (Miltenyi Biotec; 1:11), antimouse F4/80-PE (eBioscience; 1:40), antimouse CD64-APC (Biolegend; 1:200) and antimouse CD11c-BV421 (Biolegend; 1:20). Antibodies and dilutions used as isotype controls were rat IgG2b K isotype control FITC (eBioscience; 1:100), rat IgG2a K isotype control PE (eBioscience; 1:40), mouse IgG1 κ isotype control APC (Biolegend; 1:200) and Armenian hamster IgG isotype control BV421 (Biolegend; 1:40). Cells were first gated FSC-H versus FSC-A to select singlets and subsequently FSC-A versus SSC-A to select cells. Singlet cells were gated for live cells before being measured for their fluorescence from each sample fluorophore. The percentage of fluorescent cells against non-fluorescent cells was determined through Fluorescence Minus One controls.

### PD and time–kill studies

Infected mouse peritoneal exudate macrophages or BMDMs were exposed to 3-fold serial drug dilutions in RPMI 1640 medium + 10% hi-FBS, over at least six different concentrations. The highest concentrations used were 30 μM for miltefosine and 1 μM for amphotericin B. Selected experiments included an additional concentration of 3 μM for the latter. Untreated controls received medium only. Each concentration and control was tested in quadruplicate. Cultures were exposed to drug dilutions at 37°C/5% CO_2_ for 6, 12, 24, 48, 72 or 120 h. At 72 h medium and drug dilutions were refreshed. At experimental endpoints slides were fixed with 100% methanol and stained with 10% Giemsa. One hundred macrophages per well were examined microscopically and the percentage of infected macrophages calculated.

A mathematical model relating drug concentration with percentage infected cells over time was developed. The apparent intracellular concentration (*C*_I_) was predicted using a Hill-type model:

(Eqn 1) $$C_{\text{I}}=\;C_{\text{p}}\;\frac{t^{\text{γ}}}{t^{\gamma }+\;t_{50}^{\text{γ}}}\;$$

where the covariates were *C*_p_, the extracellular concentration, and *t*, the time (h), and the estimated parameters were *t*_50_, the time to reach 50% of the maximum penetration, and γ, a shape parameter. The fraction of infected cells at time *t* [*F(t)*] was then predicted from the following:

(Eqn 2) $$F\left(t\right)=F_{\text{B}}-F_{\text{B}}\frac{C_{\text{I}}^{\text{λ}}}{C_{\text{I}}^{\text{λ}}+{\mathrm{EC}}_{50}^{\text{λ}}}\;$$

where *F*_B_ is the estimated baseline fraction infected, EC_50_ is the apparent concentration to reduce the fraction infected by half, and λ is a shape (Hill) parameter. Model fitting was undertaken with NONMEM (version 7.3) and a logit transformation used to ensure predictions were between 0% and 100%. Inter-experiment variability was estimated on *F*_B_, again using a logit transformation to ensure *F*_B_ remained between 0 and 1. Improvements in model fit were assessed by looking for reductions in the objective function value upon model parameter addition. Final model evaluation used a visual predictive check whereby 1000 simulations were performed with the 2.5th, 50th and 97.5th percentiles overlaid on the raw data.

### Cellular accumulation studies and drug extraction

Miltefosine, Fungizone^®^ or AmBisome^®^ was added to infected BMDMs at selected drug concentrations in RPMI 1640 medium, supplemented with hi-FBS or BSA, in a volume of 1000 μL per well. Each concentration was tested in quadruplicate. Cultures were incubated at 37°C/5% CO_2_ and chamber slides removed from the incubator at set timepoints. Amphotericin B-treated macrophages were washed three times in 1000 μL of cold DPBS prior to the addition of 500 μL of 0.1% (v/v) formic acid in water to each well. Miltefosine-treated macrophages were washed with 3% (w/v) fatty acid-free BSA in PBS^9^ and cold DPBS prior to the addition of 500 μL of 0.1% (v/v) formic acid in water. Macrophages were lysed in 0.1% (v/v) formic acid in water at room temperature for 30 min with vigorous repeat mixing. Lysis was checked by light microscopy. Amphotericin B extraction from drug-treated cell lysates was performed by mixing 250 μL of cell lysate with 250 μL of an 84:16 (v/v) mixture of methanol/DMSO containing 200 ng/mL tolbutamide as internal standard. For extraction of miltefosine 250 μL of drug-treated cell lysate was mixed with 250 μL of acetonitrile containing 200 ng/mL tolbutamide as internal standard. After shaking for 10 min at 200 rpm at room temperature, lysate mixtures were centrifuged at 6600 rpm for 15 min at 4°C. Supernatants were transferred to 96-well plates. Supernatants and cell pellets were stored at –80°C prior to drug and protein quantification. The level of infection in *L. donovani*-infected samples was determined in untreated controls.

To calculate apparent intracellular concentrations we determined the cellular volume per mg of protein from the mean diameter of BMDMs (16.4 ± 0.8 μm) and the total protein content per well (35 μg). This gave a factor of 16 μL of cell volume per mg of cell protein for BMDMs.

### Drug quantification procedure

Drug levels in samples were quantified using reverse phase gradient elution on an Agilent 1200 HPLC with specific detection for each compound by multiple reaction monitoring on an Agilent 6410A triple quadrupole mass spectrometer (both systems from Agilent, UK). Calibration standards were prepared by spiking 237.5 μL aliquots of untreated cell lysate with 12.5 μL of drug solution at a number of different concentrations. To these were added 250 μL of the appropriate internal standard solution with further preparation and storage carried out according to the procedure described in the last section. Blank, blank + internal standard and quality control samples were included in the analyses.

### Determination of protein concentration in cell lysates

Protein concentrations in cell lysates were determined using the Pierce™ BCA Protein Assay Kit (Fisher Scientific, UK), following the manufacturer’s protocol.

### Cytotoxicity assay

*L. donovani* (strain MHOM/Sudan/09/SUKA001)-infected BMDMs were exposed to Fungizone^®^ and miltefosine in RPMI 1640 medium + 10% hi-FBS and RPMI 1640 medium with varying percentages of hi-FBS, respectively. At set timepoints (1, 6, 24, 48 or 72 h), 50 μL aliquots of supernatants were transferred to 96-well plates and the amount of lactate dehydrogenase (LDH) measured using the CytoTox 96^®^ Non-Radioactive Cytotoxicity Assay (Fisher Scientific, UK). Untreated infected BMDMs, maximum LDH release controls and no-cell controls were included. Each condition was tested in quadruplicate. Absorbance was read at 490 nm and the average values of the culture medium background subtracted from all values of experimental wells. Percentage cytotoxicity was calculated by the formula 100 × experimental LDH release (OD_490_)/maximum LDH release (OD_490_).

### Ethics

Experiments involving animals were carried out under licence in accordance with the Animals (Scientific Procedures) Act 1986 (UK Home Office Project Licences PPL70/6997 and PPL70/8207) following approval by the Animal Welfare and Ethics Review Board at LSHTM.

## Results

### Time–kill studies against intracellular L. donovani amastigotes in primary mouse macrophages

**Figure 1.**
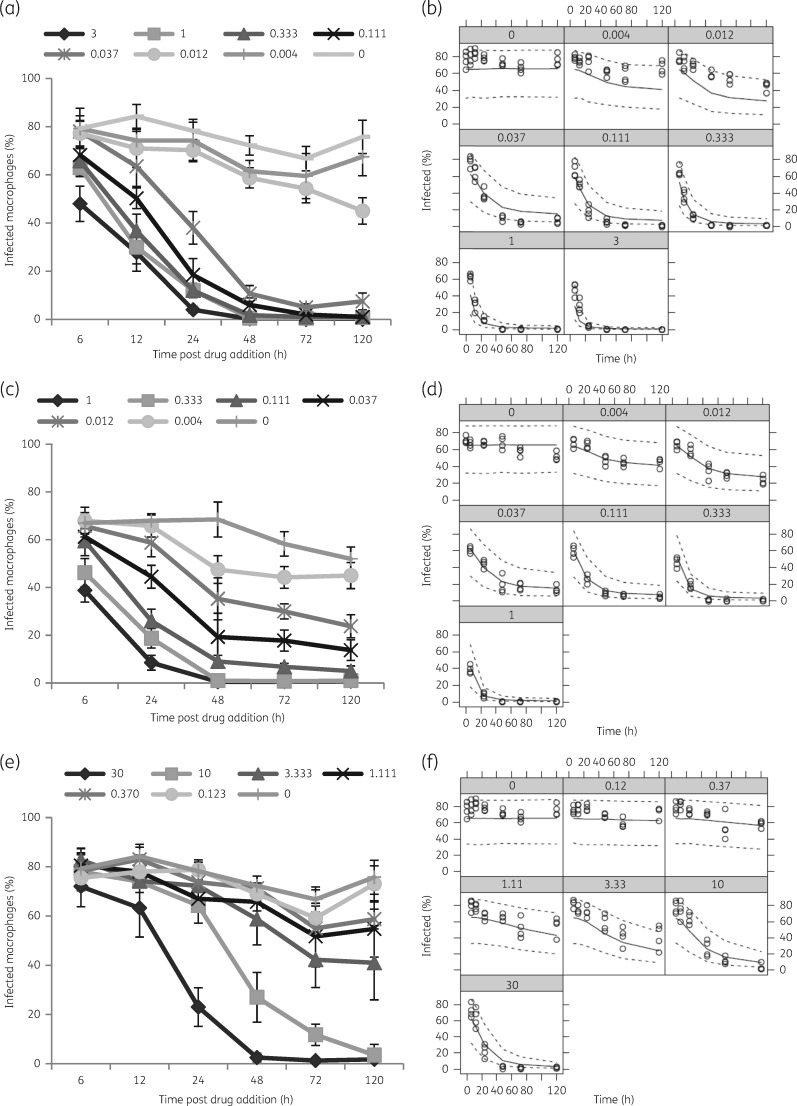
Time–kill curves for amphotericin B and miltefosine. BMDMs were infected with *L. donovani* (MHOM/Sudan/09/SUKA001) and exposed to Fungizone^®^ (a and b), AmBisome^®^ (c and d) or miltefosine (e and f) at indicated drug concentrations (µM). The percentage of infected macrophages was evaluated at the indicated timepoints. Visual predictive checks, for respective experiments, comparing the percentage of infected cells (black open circles) with model-simulated 2.5th, 50th and 97.5th percentiles of 1000 simulated datasets are shown (b, d and f). Data are shown for one of five separate experiments for miltefosine, six separate experiments for Fungizone^®^ and three separate experiments for AmBisome^®^.

Estimated EC_50_ values of amphotericin B and miltefosine were 0.65 and 1.26 μM, respectively. As the anti-leishmanial activity did not occur instantaneously, these values must be considered in relation to the apparent drug penetration time. Time to reach 50% of maximum drug penetration (*t*_50_) was 55.72 h for amphotericin B and 100.01 h for miltefosine. Model parameters are summarized in Table 1 and representative plots along with time–kill curves are shown in Figure 1.

**Table 1.** Non-linear mixed effects model parameter estimates

Outcome	Variable	Estimate (%RSE^a^)
Baseline	*F*_B_	0.65 (3.7)
Apparent intracellular concentration	*t*_50_ amphotericin (h)	55.72 (15.5)
	*t*_50_ miltefosine (h)	100.01 (40.6)
	γ amphotericin	2.55 (8)
	γ miltefosine	2.09 (17.2)
Fraction of infected cells	EC_50_ amphotericin (μM)	0.65 (24.4)
	EC_50_ miltefosine (μM)	1.26 (19.1)
	λ amphotericin	0.78 (6.3)
	λ miltefosine	1.12 (17.4)
	residual error (logit estimate)	0.26 (13)

a %RSE is the relative standard error expressed as a percentage in relation to the parameter estimates.

### Evidence-based selection of primary macrophages for cellular drug accumulation studies

**Figure 2.**
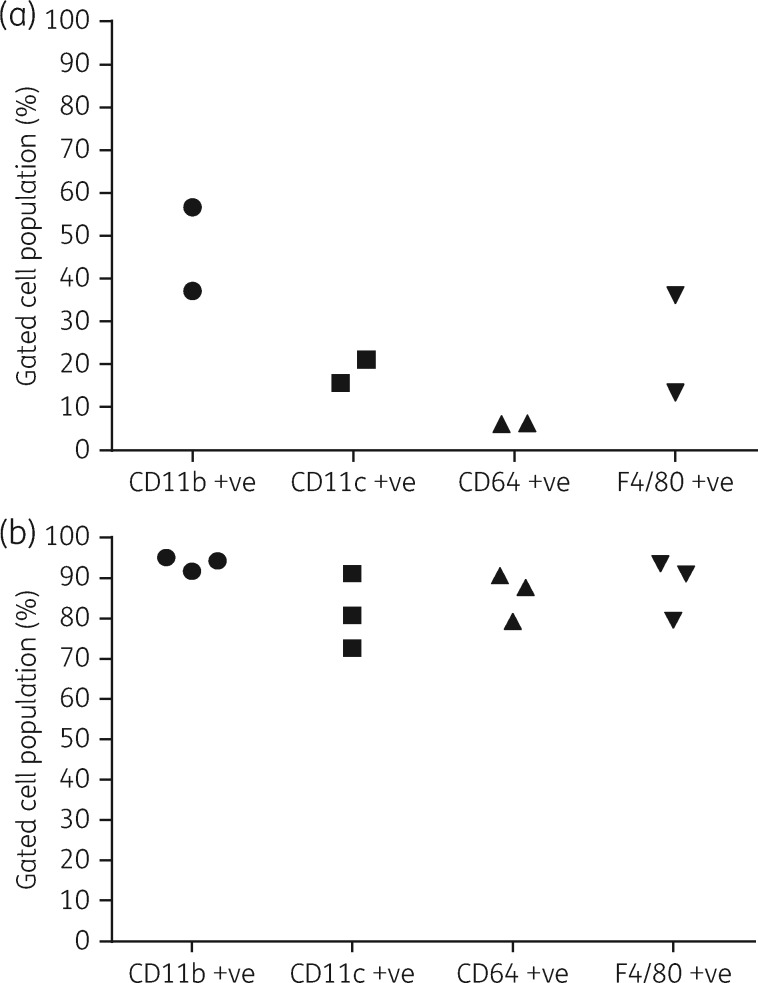
Expression levels of different surface markers on PECs and BMDMs. Each data point represents the average percentage of gated cell populations (*n* = 4) for indicated markers in separate experiments in PECs (a) and BMDMs (b). Standard deviations were ≤1.5 for PECs and ≤4.4 for BMDMs and, thus, they are not shown in the plots. The percentages of live cells in repeat experiments were 79.2 ± 4.4% and 79.3 ± 5.3% in PECs and 85.7 ± 0.8% and 89.8 ± 2.0% in BMDMs.

Expression of selected cell surface markers was lower and more heterogeneous in PECs than in BMDMs. In repeat experiments F4/80 was expressed by ≤36.1 ± 0.2% of PECs and ≥79.6 ± 0.7% of BMDMs, CD11b was expressed by ≤56.7 ± 0.5% of PECs and ≥87.1 ± 1.2% of BMDMs, CD11c was expressed by ≤21.0 ± 1.5% of PECs and ≥72.7 ± 0.3% of BMDMs and CD64 was expressed by ≤6.2 ± 0.4% of PECs and ≥79.4 ± 0.4% of BMDMs (Figure 2). BMDMs were chosen as host cells in cellular accumulation studies.

### Cellular accumulation of amphotericin B in L. donovani-infected BMDMs over time

**Figure 3.**
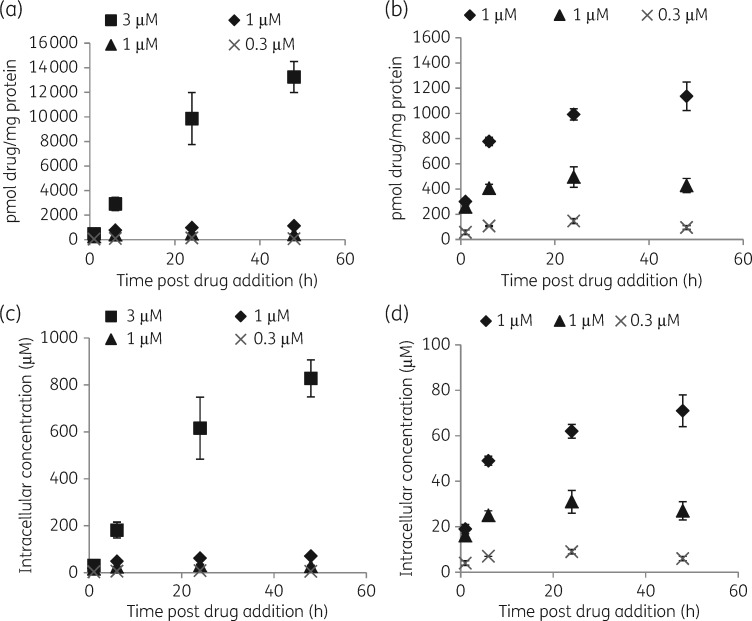
Concentrations of cell-associated amphotericin B over time following exposure to Fungizone^®^. Experiments were carried out in RPMI 1640 medium + 10% hi-FBS. Squares represent cellular drug association at an exposure to 3 µM amphotericin B, diamonds and triangles represent cellular drug association at an exposure to 1 µM amphotericin B and crosses represent cellular drug association at an exposure to 0.3 µM amphotericin B. Data represented by diamonds and squares were obtained in the same experiment, as well as data represented by triangles and crosses. (a and c) Data for all drug concentrations. (b and d) Data for the lower two drug concentrations. Data points represent the means (*n *=* *4) and the error bars represent the standard deviations. Data are shown for one of two or three separate experiments.

Infected BMDMs were exposed to Fungizone^®^ in RPMI 1640 medium + 10% hi-FBS at concentrations exerting >90% intracellular parasite kill (Figure 1). The highest increase in cellular drug concentration was observed between 1 and 6 h (5–6-fold at 3 μM and 1.6–2.6-fold at 1 μM in repeat experiments). At 3 μM amphotericin B cellular concentrations also increased between 6 and 24 h (2–3.4-fold), and between 24 and 48 h (1.3–1.6-fold). At 1 μM amphotericin B cellular drug concentrations increased between 6 and 24 h (1.2–1.9-fold), but remained at similar levels between 24 and 48 h (0.9–1.1-fold differences). At an exposure to 0.3 μM amphotericin B, differences in cellular drug concentrations were 1.3–1.8-fold between 1 and 6 h, 1.3–2.2-fold between 6 and 24 h and 0.7–1.2-fold between 24 and 48 h (Figure 3).

**Figure 4.**
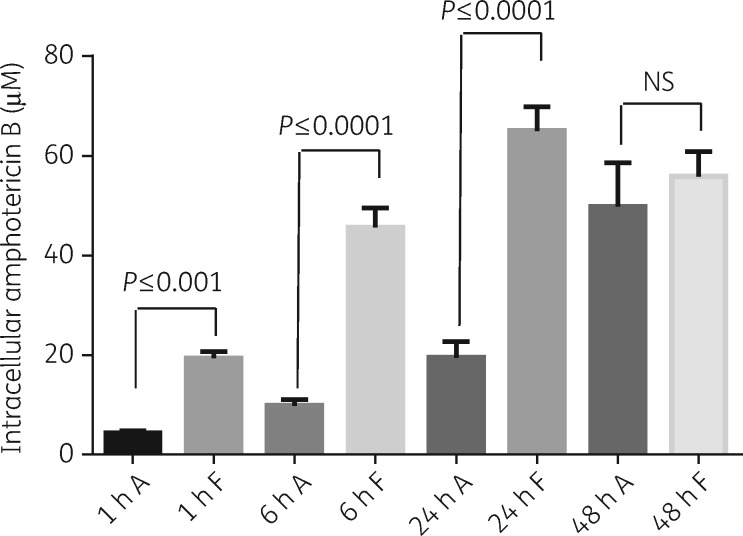
Cellular concentrations of amphotericin B over time following exposure of infected BMDMs to AmBisome^®^ (A) or Fungizone^®^ (F). Experiments were carried out in RPMI 1640 medium + 10% hi-FBS. Columns represent the means (*n *=* *4) and the error bars represent the standard deviations. Data are shown for one of two separate experiments. Statistical significance, defined as *P* < 0.05, was evaluated by one-way analysis of variance, assuming Gaussian distribution, and Sidak’s multiple comparisons test (GraphPad Prism 6). NS, non-significant.

When infected BMDMs were exposed head-to-head to Fungizone^®^ and AmBisome^®^ at amphotericin B concentrations of 1 μM, maximum cellular drug concentrations were reached within 24 h following incubation with Fungizone^®^. In contrast, cellular amphotericin B concentrations increased over the whole period following incubation with AmBisome^®^. Cellular drug concentrations were higher following exposure to Fungizone^®^ compared with AmBisome^®^ at all timepoints tested (Figure 4). This difference was statistically significant at 1, 6 and 24 h in repeat experiments and at 48 h in one experiment. Tabulated results are provided in Table S1 (available as Supplementary data at *JAC* Online).

### Cellular accumulation of miltefosine in L. donovani-infected BMDMs over time

**Figure 5.**
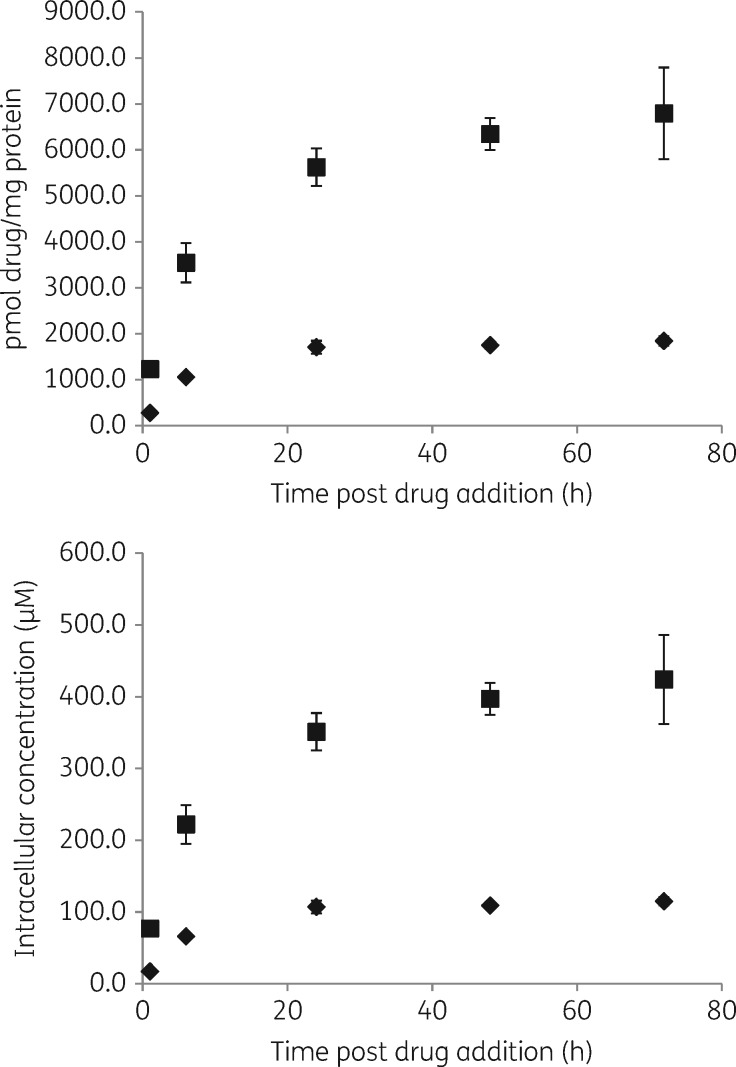
Concentrations of cell-associated miltefosine over time. Experiments were carried out in RPMI 1640 medium + 10% hi-FBS. Squares represent cell association at an exposure to 30 µM miltefosine and diamonds represent cell association at an exposure to 10 µM miltefosine. Data points represent the means (*n* = 4) and the error bars represent the standard deviations. Data are shown for one of two separate experiments.

Infected BMDMs were exposed to miltefosine in RPMI 1640 medium + 10% hi-FBS at concentrations exerting >90% intracellular parasite kill (Figure 1). At both concentrations, 30 and 10 μM, respectively, the highest increase in cellular drug concentrations was observed between 1 and 6 h (3.2- and 2.9-fold at 30 μM and 3.4- and 3.9-fold at 10 μM in repeat experiments), with a further increase between 6 and 24 h (1.3- and 1.6-fold at 30 and 10 μM). Concentrations remained similar between 24 and 48 h (in repeat experiments), and 48 and 72 h (in one experiment), with fold differences between 0.9 and 1.1 (Figure 5). Tabulated results are provided in Table S2.

### Profiling toxicity of amphotericin B and miltefosine against L. donovani-infected BMDMs over time

**Figure 6.**
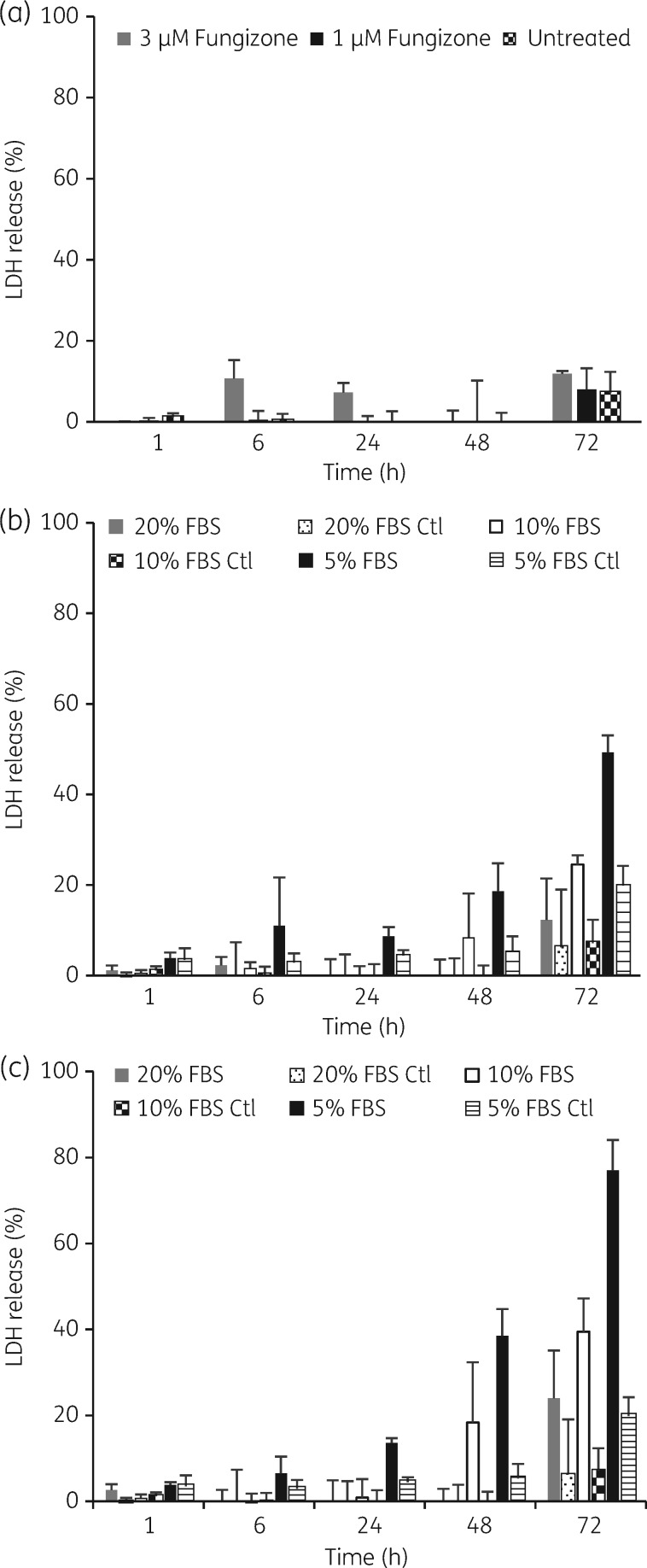
Cytotoxicity of amphotericin B and miltefosine against *L. donovani*-infected BMDMs. Results are expressed as percentage of total LDH obtained from completely lysed cells. LDH release was measured after incubation of cells (a) in medium with 10% hi-FBS at amphotericin B concentrations of 3 and 1 µM, (b) in medium with 5%, 10% or 20% hi-FBS at miltefosine concentrations of 30 µM or (c) in medium with 5%, 10% or 20% hi-FBS at miltefosine concentrations of 10 µM. Columns represent the means (*n *=* *4) and the error bars represent the standard deviations. Data are shown for one of two separate experiments. Ctl, control.

LDH release was measured as an indicator of plasma membrane damage. For amphotericin B the highest LDH release observed was 11% and 12% after 6 and 72 h of exposure to a concentration of 3 μM in medium containing 10% hi-FBS. LDH release in untreated controls was ≤7% (Figure 6a). For miltefosine LDH releases of 40%, 25% and 18% were observed after 72 h of exposure to 30 and 10 μM and 48 h exposure to 30 μM in medium containing 10% hi-FBS. LDH release in all other drug-treated samples and untreated controls was ≤10%. Increasing hi-FBS in the assay medium to a concentration of 20% decreased LDH releases to 24% and 12% after 72 h of exposure to 30 and 10 μM miltefosine. Lowering the concentration of hi-FBS to 5% induced LDH release at earlier timepoints and increased its magnitude compared with medium containing 10% and 20% hi-FBS. LDH release in untreated controls reached a maximum of 20% after 72 h of exposure to medium with 5% hi-FBS, but was <10% at all other timepoints (Figure 6b and c).

### Effect of varying hi-FBS and BSA concentrations in medium on the cellular accumulation of miltefosine

**Figure 7.**
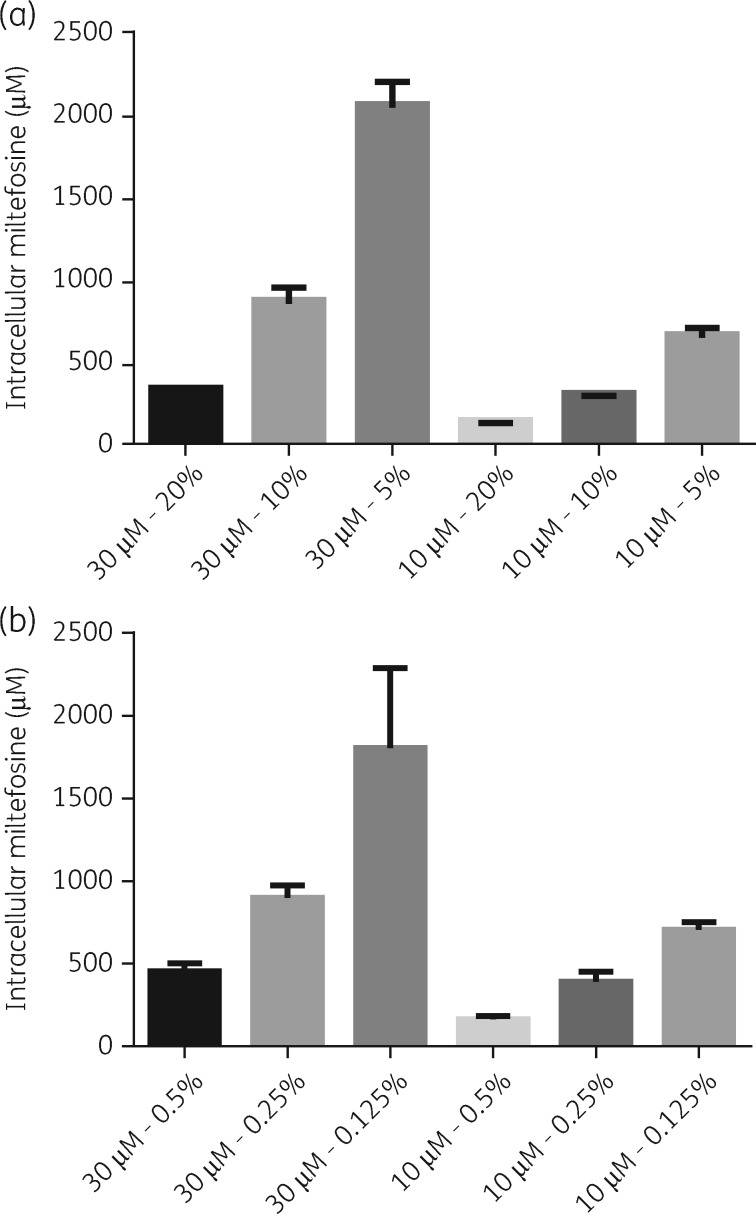
Effect of different hi-FBS and BSA concentrations on cell-associated miltefosine. Medium was supplemented with 20%, 10% or 5% hi-FBS (a) or 0.5%, 0.25% or 0.125% of BSA (b) and cells exposed to drug dilutions for 24 h. Columns represent the mean (*n *=* *4) and error bars standard deviations. Data are shown for one of two separate experiments for each condition.

Assay medium was supplemented either with 20%, 10% and 5% hi-FBS, or 0.5%, 0.25% and 0.125% BSA (equivalent albumin concentrations present in 20%, 10% and 5% hi-FBS) and cellular drug levels in *L. donovani*-infected BMDMs measured after 24 h of exposure to 30 and 10 μM miltefosine. Decreased cellular miltefosine levels were observed with increasing protein concentrations in medium in all experiments (Figure 7).

### Effect of varying host cell numbers on the cellular accumulation of miltefosine

**Figure 8.**
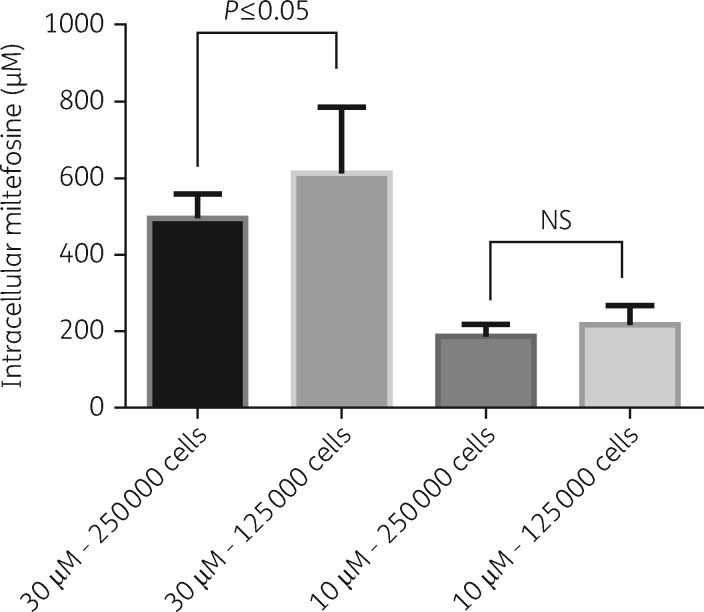
Effect of host cell numbers on cell-associated miltefosine. Columns represent the means (*n* = 8) and the error bars represent the standard deviations. Data are shown as combined data from two separate experiments. Statistical significance, defined as *P* < 0.05, was evaluated by one-way analysis of variance, assuming Gaussian distribution, and Sidak’s multiple comparisons test (GraphPad Prism 6). NS, non-significant.

The effect of host cell density on cell-associated drug concentrations was investigated after 24 h of exposure of infected BMDMs to two different concentrations of miltefosine in RPMI 1640 medium + 10% hi-FBS. In one experiment a statistically significant higher (*P* ≤ 0.01) cellular concentration was observed when 125 000 cells/well were exposed to 30 μM miltefosine compared with 250 000 cells/well. However, this difference was not reproduced in a repeat experiment and no difference in cellular concentrations between the two plating densities was observed at an exposure to 10 μM miltefosine (Figure 8).

## Discussion

*In vitro* evaluation of anti-leishmanial drug activity has been limited to point estimates of the PD effect, through determination of EC_50_ and EC_90_ values at specified timepoints. Recently, time–kill experiments for standard anti-leishmanial drugs aimed to identify the minimal exposure time needed to eliminate viable intracellular amastigotes at selected drug concentrations.^10^ Here we developed a PD modelling approach, where apparent drug concentrations have been used to estimate EC_50_ values. This approach allowed characterization of drug effects over the whole evaluation period, with EC_50_ values indicating higher potency of amphotericin B over miltefosine. Modelling of *in vitro* time–kill experiments has been applied to other anti-infectives, including antibacterials^11^ and antifungals,^12^ but is a novel approach for anti-leishmanials. In PK/PD models, it is well known that homogenate-derived drug concentrations rarely relate to meaningful antimicrobial activity concentrations, as drugs are rarely evenly distributed between compartments and subcellular organelles.^13^ This may explain why the sigmoidal shape empirically best described the apparent intracellular concentrations, which were based on antimicrobial activity inferred from a decreasing percentage of infected cells.

Amphotericin B and miltefosine display similar activities against intracellular *L. donovani* amastigotes in peritoneal exudate macrophages and BMDMs,^8^ supporting the use of both cell types in PD studies. However, to provide a rational approach for the selection of one cell type in drug accumulation studies we characterized cells obtained from peritoneal exudate and 6 day differentiated macrophages from bone marrow through their expression of selected surface markers, including F4/80, CD11b, CD11c and CD64.^14–16^ The lower percentage of PECs expressing these markers compared with BMDMs may be due to the harvest of PECs 1 day after the inflammatory stimulus. A change of subsets of immune cells over time has been reported for PECs, following a thioglycolate stimulus, with neutrophils outnumbering macrophages and lymphocytes 1 day after stimulation.^16^

Previous studies have characterized amphotericin B uptake into CHO and J774 cells^17^^,^^18^ and into *L. donovani*-infected and -uninfected differentiated THP-1 cells.^19^ However, in these studies cells were exposed to significantly higher amphotericin B concentrations and for shorter periods of time than used to demonstrate anti-leishmanial efficacy. Here we measured cellular drug accumulation at concentrations and timepoints selected based on time–kill curves and PD effects. The kinetics of the cellular accumulation of amphotericin B were concentration- and formulation-dependent. Exposure to Fungizone^®^ at 3 μM amphotericin B resulted in a steeper concentration versus time curve than exposure to 1 and 0.3 μM amphotericin B. Dilution of Fungizone^®^ <5 μM leads to the loss of deoxycholate from the mixture and complete dissociation.^17^ Free amphotericin B at <1 μM is predominantly monomeric and its aggregation state affects drug interaction with membranes.^20^ Endocytosis has been demonstrated as the route of internalization of amphotericin B into CHO cells with both rate and drug distribution along the endocytic pathway concentration-dependent.^18^ The lower cellular drug accumulation following incubation with AmBisome^®^ compared with Fungizone^®^ is consistent with previous observations^17^^,^^19^ and it is known that the lipids in AmBisome^®^ slow the rate of transfer of amphotericin B molecules to cell membranes.^21^

Highly effective amphotericin B concentrations caused minimal membrane damage. In contrast, effective miltefosine concentrations caused LDH release, indicating a narrower *in vitro* therapeutic window of miltefosine over amphotericin B. Miltefosine induced membrane damage in mammalian cells in a concentration-, time- and serum-dependent manner, possibly through its interaction with membrane proteins and induction of structural changes.^22^ A number of studies have investigated cell uptake and membrane interactions of miltefosine^23–25^ and an efflux transporter has been identified in human macrophages.^26^ In KB cells miltefosine was located in the plasma membrane and, to a greater extent, intracellular membranes, with a rapid distribution between plasma and intracellular membranes.^25^ We noted that, at an extracellular concentration of 10 μM miltefosine, the timepoint at which maximum total cellular drug concentrations were reached preceded the timepoint at which maximum parasite killing was observed. Rapid drug distribution throughout the cell would rule out drug distribution as an explanation and support a mode of time-dependent killing for miltefosine.

Miltefosine binds to plasma proteins from rats, dogs and humans (www.accessdata.fda.gov, application number 204684Orig1s000), with albumin identified as the major protein involved in binding in human serum.^27^ As hypoalbuminaemia is observed in human and experimental VL^28^^,^^29^ we investigated the effect of varying albumin concentrations in assay medium on cellular miltefosine levels and membrane damage. Although protein binding is species-specific,^30^ the inverse relationship between serum/albumin concentration in medium and LDH release suggests that membrane damage is caused by unbound miltefosine. In addition, the inverse relationship between the serum/albumin concentration in medium and cellular drug accumulation supports the model in which unbound miltefosine interacts with plasma membranes of host macrophages and is the predominant species to be internalized.^31^ A lower threshold of cytotoxicity in the absence of serum has previously been reported for amphotericin B.^17^ Another feature of human and experimental VL is the accumulation of mononuclear phagocytic cells in infected tissues.^32^^,^^33^ In *L. donovani*-infected spleens in BALB/c mice the percentage and total number of red pulp macrophages increase by 1.9- and 6.5-fold, respectively, within 35 days of infection.^34^ However, within the two different host cell densities used here no clear relationship between host cell number and drug accumulation emerged.

In conclusion, we have developed a novel approach to investigate *in vitro* PD and cellular anti-leishmanial drug accumulation over time and have applied this to investigate how host factors impact on drug accumulation. The work presented here provides a model for the characterization of new compounds and drug candidates.

## Supplementary Material

Supplementary Data
