# Supplementary material for: Pharmacodynamics and cellular accumulation of amphotericin B and miltefosine in Leishmania donovani-infected primary macrophages
Source: J Antimicrob Chemother. 2018 Feb 28;73(5):1314–23. doi: 10.1093/jac/dky014 (PMC5909632; doi:10.1093/jac/dky014)
Supplement: Supplementary Data [file dky014_supplementary_data.docx]

**Supplementary data**

**Table S1.**

| **Drug** | **Conc. [µM]** | **Parasite strain** | **Expt.** | **Time post drug addition (hrs)** | | | |
| --- | --- | --- | --- | --- | --- | --- | --- |
|  |  |  |  | **1** | **6** | **24** | **48** |
|  |  |  |  | **Cell associated drug [pmol drug / mg protein] +/- SD** | | | |
| **Fungizone®** | **3** | SUKA001 | 1 | 488 +/- 52 | 2 918 +/- 546 | 9 862 +/- 2 114 | 13 246 +/- 1 266 |
| **Fungizone®** | **1** | SUKA001 | 1 | 301 +/- 24 | 778 +/- 32 | 992 +/- 44 | 1 136 +/- 113 |
| **Fungizone®** | **3** | HU3 | 2 | 687 +/- 143 | 3 459 +/- 220 | 6 877 +/- 546 | 10 710 +/- 307 |
| **Fungizone®** | **1** | HU3 | 2 | 299 +/- 20 | 607 +/- 31 | 1 178 +/- 81 | 1 010 +/- 91 |
| **Fungizone®** | **1** | SUKA001 | 3 | 258 +/- 23 | 406 +/- 31 | 495 +/- 82 | 428 +/- 57 |
| **Fungizone®** | **0.3** | SUKA001 | 3 | 57 +/- 18 | 107 +/- 4 | 146 +/- 19 | 95 +/- 15 |
| **Fungizone®** | **0.3** | SUKA001 | 4 | 65 +/- 14 | 82 +/- 11 | 175 +/- 4 | 215 +/- 54 |
| **AmBisome®** | **1** | SUKA001 | 5 | <LLOQ | 97 +/- 7 | 231 +/- 16 | 354 +/- 63 |
| **Fungizone®** | **1** | SUKA001 | 5 | 289 +/- 58 | 505 +/- 119 | 651 +/- 43 | 481 +/- 46 |
| **AmBisome®** | **1** | SUKA001 | 6 | <LLOQ | 157 +/- 20 | 312 +/- 52 | 797 +/- 141 |
| **Fungizone®** | **1** | SUKA001 | 6 | 310 +/- 23 | 731 +/- 62 | 1040 +/- 79 | 894 +/- 80 |
|  |  |  |  |  |  |  |  |
|  |  |  |  | **Intracellular drug [µM] +/- SD** | | | |
| **Fungizone®** | **3** | SUKA001 | 1 | 31 +/- 3 | 182 +/- 34 | 616 +/- 132 | 828 +/- 79 |
| **Fungizone®** | **1** | SUKA001 | 1 | 19 +/- 2 | 49 +/- 2 | 62 +/- 3 | 71 +/- 7 |
| **Fungizone®** | **3** | HU3 | 2 | 43 +/- 9 | 216 +/- 14 | 430 +/- 34 | 669 +/- 19 |
| **Fungizone®** | **1** | HU3 | 2 | 19 +/- 1 | 38 +/- 2 | 74 +/- 5 | 63 +/- 6 |
| **Fungizone®** | **1** | SUKA001 | 3 | 16 +/- 1 | 25 +/- 2 | 31 +/- 5 | 27 +/- 4 |
| **Fungizone®** | **0.3** | SUKA001 | 3 | 4 +/- 1 | 7 +/- 0 | 9 +/- 1 | 6 +/- 1 |
| **Fungizone®** | **0.3** | SUKA001 | 4 | 4 +/- 1 | 5 +/- 1 | 11 +/- 1 | 13 +/- 3 |
| **AmBisome®** | **1** | SUKA001 | 5 | <LLOQ | 6 +/- 0 | 14 +/- 1 | 22 +/- 4 |
| **Fungizone®** | **1** | SUKA001 | 5 | 18 +/- 4 | 32 +/- 7 | 41 +/- 3 | 30 +/- 3 |
| **AmBisome®** | **1** | SUKA001 | 6 | <LLOQ | 10 +/- 1 | 19 +/- 3 | 50 +/- 9 |
| **Fungizone®** | **1** | SUKA001 | 6 | 19 +/- 1 | 46 +/- 4 | 65 +/- 5 | 56 +/- 5 |

**Table S2.**

| **Drug** | **Conc. [µM]** | **Parasite strain** | **Expt.** | **Time post drug addition (hrs)** | | | | |
| --- | --- | --- | --- | --- | --- | --- | --- | --- |
|  |  |  |  | **1** | **6** | **24** | **48** | **72** |
|  |  |  |  | **Cell associated drug [pmol drug / mg protein] +/- SD** | | | | |
| **Miltefosine** | **30** | SUKA001 | 1 | 2 400 +/- 637 | 7 633 +/- 537 | 10 114 +/- 1 037 | 9 432 +/- 477 | 11 865 +/- 2 527 |
| **Miltefosine** | **10** | SUKA001 | 1 | 759 +/- 93 | 2 569 +/- 284 | 3 233 +/- 336 | 3 081 +/- 225 | 5 174 +/- 397 |
| **Miltefosine** | **30** | SUKA001 | 2 | 1230 +/- 35 | 3545 +/- 428 | 5622 +/- 410 | 6345 +/- 350 | 6792 +/- 999 |
| **Miltefosine** | **10** | SUKA001 | 2 | 279 +/- 19 | 1058 +/- 25 | 1705 +/- 141 | 1752 +/- 86 | 1844 +/- 99 |
|  |  |  |  |  |  |  |  |  |
|  |  |  |  | **Intracellular drug [µM] +/- SD** | | | | |
| **Miltefosine** | **30** | SUKA001 | 1 | 150 +/- 40 | 477 +/- 34 | 632 +/- 65 | 590 +/- 30 | 742 +/- 158 |
| **Miltefosine** | **10** | SUKA001 | 1 | 47 +/- 6 | 161 +/- 18 | 202 +/- 21 | 193 +/- 14 | 323 +/- 25 |
| **Miltefosine** | **30** | SUKA001 | 2 | 77 +/- 2 | 222 +/- 27 | 351 +/- 26 | 397 +/- 22 | 424 +/- 62 |
| **Miltefosine** | **10** | SUKA001 | 2 | 17 +/- 1 | 66 +/- 2 | 107 +/- 9 | 109 +/- 5 | 115 +/- 6 |
